# Social and cultural efficacies of medicines: Complications for antiretroviral therapy

**DOI:** 10.1186/1746-4269-2-48

**Published:** 2006-11-07

**Authors:** Sjaak van der Geest, Anita Hardon

**Affiliations:** 1 Medical Anthropology, University of Amsterdam, The Netherlands

## Abstract

Using ethnographic examples of medicine use, prescription, distribution and production, the authors argue that social and cultural effects of pharmaceuticals should be taken into account. Non-medical effects deeply influence the medical outcome of medicine use. Complications around the advent of anti-AIDS medicines in poor countries are taken as a point in case. The authors are medical anthropologists specialised in the social and cultural analysis of pharmaceuticals.

## Background

As anthropologists studying medicines we look at medicines as social and cultural phenomena. We do not ignore or overlook their therapeutic function, but want to draw attention to aspects that usually *are *overlooked: their social, cultural, economic, religious and emotional effects. Medical and non-medical effects are not unrelated, however; together they form the total drug effect. They may reinforce each other (placebo effect) or work against each other (nocebo effect).

Some years ago, we [1,2] used a popular anthropological metaphor to capture the social and cultural dimension of medicines, Appadurai's concept of 'Social Life of Things' [3]. Things acquire meaning, when they enter into the life of people. There begins their 'social life'. Nothing (no-thing) coincides completely with its material qualities. Every-thing transcends its rustic entity and assumes social, cultural, psychological (etc.) roles in the world of human beings. Things are bound to be semiotic (having symbolic meanings). That semiotic quality is likely to grow if objects are more valuable.

Medicines are a good example of valuable things that assume a wide variety of meanings, far beyond their material (chemical) properties. We have tried to illustrate this in our book by looking at medicines in the hands of different actors: consumers, such as patients and caretakers, providers (pharmacists, drug vendors, physicians), producers and what we could call 'paper workers'. Let us look more closely at some of these actors.

## Mothers in Manila

In one chapter we describe how mothers in a poor area of Manila give medicines to their children when they suffer from coughs or colds [4]. After discussing the mothers' ideas about coughs and colds and how they should be treated (quite different from biomedical knowledge), the chapter points at ideas that underlie these narratives of causation and therapy; that is concerns about the vulnerability of children. Children need protection and parents should be responsible. If children are ill often, people tend to talk about their mothers as negligent. Cough, as Tan observes, is considered undesirable and blameworthy in the urban poor communities. He cites a young mother who describes this graphically: '..if the cough is continuous, (the child's) father is disturbed. When he comes back from work, and is hot headed, then he hears the child coughing... Because I am the mother, if the child is sick, I am blamed.' [5]. Tan argues that it is not surprising that the manufacturers of cough and cold medicines emphasize a hacking dry cough in their advertising. The dry cough conveys not just the image of lungs bursting from the strain, but also the potential of social disruption.

The idea that children usually recover without medicines has no chance in such a situation. The social context demands the use of medicines. The effects of those medicines are therefore manifold:

• They may help to cure the child faster.

• They confirm to the mother that she is a good mother.

• They send a message to the child that the mother cares.

• They communicate that same message to the husband, neighbours and others.

• And all these messages together reinforce the health restoring effect on the sick child.

## Women in distress

In her study 'Rituals of Silence' Haafkens [6] examines the use of benzodiazepines – medicines prescribed for mental distress – by women in the Netherlands. She describes how the medicines not only make it possible for women to live on with their mental health problems, but also provide society with a means to control anxiety and stress. The ambiguous relation between self-control of female distress and medicalised social control of life problems is the main theme of another chapter in our book.

On the one hand, women use benzodiazepines like other medicines to enhance the quality of their individual lives. Medicines liberate them from bodily discomfort, and give them means to control natural bodily processes such as conception, menstruation and menopause. Medicines are part of day-to-day body regimes, in which women strive to fulfill societal expectations of work capability, appropriate fertility, attractive appearance, and mental stability.

On the other hand, benzodiazepines and other medicines also function as a medical means of social control. In modern societies, where medicine has replaced religion as a dominant moral ideology and social control institution, more and more of everyday life has come under medical dominion, influence and supervision, a process known as medicalisation.

Around 1990, at the time of Haafkens' study, about one in every seven women had a prescription for benzodiazepines filled annually in the Netherlands; men received such prescriptions much less often. Dutch medical guidelines acknowledged that the drugs were not as safe as initially thought, and recommended that the duration of benzodiazepine use be limited to one to two weeks, a few months at the most. Despite this advice, long-term use of benzodiazepines remained relatively common in the Netherlands, affecting an estimated three percent of the Dutch population.

The efficacy of the medicines, therefore, is ambiguous. For the women who use them, the medicines are indispensable to stay in control of their lives. At the same time, many of those using them feel that the medicines have taken control of *them*. They would like to stop but cannot. The medicines are empowering as well as disempowering.

## Prescribing doctors

The third example is taken from research in Sri Lanka. Lisbeth Sachs, a Swedish anthropologist, did fieldwork in a rural health unit. She followed 50 patients before, during and after their encounter with the doctor.

Sachs made a remarkable discovery. Doctors and patients hardly communicated with one another. The practitioner did not hear the patient's complaint and the patient did not understand the doctor's diagnosis but – miraculously – both parties felt satisfied about the encounter, which always ended in a prescription.

In the interview before the encounter with the doctor, patients described their problems in Ayurvedic terms, mentioning symptoms like heat escaping from the body, dry phlegm, yellow vomit and loss of semen, but during the consultation many of these symptoms were never mentioned. Instead of 'burning feeling' or 'heat behind the eyes' the patient would just say 'fever' and instead of 'too much phlegm', he would say 'cough'. The doctor who had very little, if any, time for communication would immediately make a biomedical diagnosis and write a prescription which was '...committed to paper without any evidence ...of an examination, few spoken words and little, if any, eye or body contact' [7]. Then the next patient was called.

Interestingly, the patients usually did not feel neglected or misunderstood; their respect for the doctor's expertise made them accept the doctor's verdict as the right decision and the doctor in turn, felt that she had done a good job. Sachs writes:

The mutual confidence of practitioners and patients in the medicines helps prevent the misunderstanding of each other's beliefs from being uncovered. This confidence imbues the medicines with a magic, symbolic aura, giving the practitioners as well as the patients a feeling that they contribute in their way to solving the acute health problem.... Within the therapeutic encounter then, there exists two different systems of knowledge through which the effectiveness of these medicines is integrated. The contradictory symbolic meanings of the medicines remain unrevealed, allowing patients and practitioners to communicate in a satisfactory manner [7].

Sachs describes prescribing medicines as an act which creates the illusion of communication and which results in satisfaction among both patients and doctors. It reveals five facets of prescription. Firstly, prescribing medicines is a way of dealing with uncertainty; often the doctor and the patient do not know the exact nature of the complaint nor what to do about it. Yet prescribing a medicine alleviates that uncertainty and gives both parties a feeling of having dealt with the problem.

Secondly, prescribing is a token of concern on the part of the doctor. It shows that the doctor is a good doctor. Far from being an anonymous commodity, the medicine becomes a symbolic representation of the person of the doctor. Such a positive reception of medicine creates an ideal condition for recovery.

Thirdly, prescribing is the prerogative of doctors. It is an act in which they express and reaffirm their authority over patients and over professional colleagues such as nurses and pharmacists who are not supposed to write a prescription.

Fourthly, the prescription itself, a tiny hand-written document, deserves our attention. It shares with other types of scripts the capacity to objectify, concretise and visualise. The prescription is one of the most cherished paraphernalia of the doctor expressing his superior knowledge and authority, but as an inscription, it is highly significant for the patient as well.

Finally, for the doctors in Sri Lanka, who have hardly any time to lose due to the large number of patients, prescribing is a ritual and effective way to manage the length of the consultation firmly by cutting it off and at the same time letting the patient leave in a positive mood.

## Manufacturers of medicines

Few anthropologists have succeeded in doing research in the laboratories and offices of the pharmaceutical industry. One who did manage to do so is Maarten Bode [8] who studied the culture of production and sale of Ayurvedic and Unani medicines in India. Bode shows that these medicines become symbols of an ambivalent Indian cultural identity. On the one hand they are presented as being rooted in ancient knowledge, which is very different from Western traditions; on the other hand, these medicines in their present-day form (capsules, pills, etc) are signs of modernity.

Bode also points out that the pharmaceutical companies he studied not only produce *medicines*, but also *ideas *about medicines in the form of quasi-scientific knowledge. These ideas have mainly two functions: they confirm to the producers themselves that their products are indeed wholesome and effective and they establish scientific claims that should boost their products in international competition. ' Western' pharmaceutical companies may not act very differently in this respect. They sell the disease before selling the drugs, as Healy [9] remarked in a study of a company producing medicines against panic attacks. The company exerted its influence on academic writing and sponsored scientific conferences. Healy suggests that a large, but of course unknown, percentage of the scientific output in scientific journals may now be ghost-written by agents of the industry. Bode, in his Ayurvedic study, emphasises the ritual purpose of scientific testing. The tests do not stand the test of scientific rigour but serve promotional purposes.

## Policymakers

The term 'policymakers' suggests that these people make policy. This would, with regard to medicines, mean that they plan and organise a fair and efficient distribution of medicines. We prefer a less optimistic, somewhat ironic, definition of policymakers. We see them first of all as producers of paper documents that *speak *about efficient and equitable medicine distribution. The culture of policymaking is mainly a paper culture. The task of a policymaker is to write texts before a certain deadline and get them accepted by those who carry political authority. Medicines are for them words on paper. About one and a half decades ago, a team of researchers was to evaluate the WHO's Essential Drugs Programme in a selection of developing countries. The team did not investigate whether essential medicines were indeed available and used at all levels of the selected societies. The investigators limited themselves to the question whether and how frequently the term 'essential medicines' occurred in relevant government documents.

The real medicines play only a secondary role; they come after their paper metonyms. A *de facto *fair and steady distribution of medicines would be a significant political achievement and lend credibility to a government. It would be one of the most convincing proofs of good governance: a government caring for the health of its population as a mother for her children. Medicines are often seen as the hard core of good health care, so one would expect policymakers to grab that chance and make sure that people get their medicines. It happens amazingly little in low-income countries. During research in Cameroon Van der Geest found that shortages of medicines in public hospitals and health centres had become proverbial tokens of the government's inefficiency, lack of commitment and corruption. The policymakers did not seem to care. Their paper work was perfect [10].

Finally, we must acknowledge that planning medicine distribution is not so easy. Medicines, as we have seen, have commercial, social and other values, which draw them out of the control of medical planning [11]. Medicines lead their own social lives and do not listen to the directives of the Ministry of Health. No policymaker could prevent that a laxative became the most popular contraceptive in Ghana when Van der Geest did research in that country. Most were not even aware of it. And no policymaker will be able to control the distribution of anti-AIDS medicines in Africa today.

## Anthropologists

For anthropologists too medicines are first of all a topic to write about in books and articles that are read and commented upon by colleagues and that are presented and discussed at conferences. For anthropologists, medicines provide a fascinating case to study the working of culture. In the social lives of medicines all themes that interest anthropologists seem to come together: surviving strategies, social relationships, power and inequality, poverty and affluence, belief, economic rationality, embodiment, symbolism and globalisation, to mention only a few. Paraphrasing Lévi-Strauss we can say that medicines are good to write about.

But medicines also allow anthropologists to leave their paper world and apply the results of their research in concrete situations of health care. Medicines have intrigued anthropologists, and fuelled their imagination, yet ideas and practices around medicines were not simply data to be analysed. They were concerned about health problems and they felt that what they had learned had implications for health professionals and policy makers. But they did not necessarily think that their knowledge should be used simply to facilitate their agendas. Rather they wanted to use it to interrogate public health paradigms and professional practice, to draw attention to blind spots and biases, including the neglect of consumer agency. They also wanted, at times, to go beyond analysis and critique, to engage themselves in concrete projects of action.

## Future social science research on medicines

Let us now propose a few themes for future social science research on medicines.

• We should pay more attention to the non-medical meanings and effects of medicines, as we have just attempted to do. Even if we are ultimately concerned about a just provision and good use of medicines in health care, we should study how social and other meanings of medicines impinge on the quality of provision and use. Medicines mean different things and serve different interests to different people in different situations. No prescriber or policymaker can afford to overlook that complexity of medicines.

• We should be particularly attentive to the economic reality of medicines. Medicines are commodities, and extremely lucrative ones at that. If we want to understand the ways of medicines, we should study the directions of money. Recent discussions and developments regarding the distribution of anti-AIDS medicines to poor countries underscore how true this is [12,13].

• We want to emphasise the need for studying how our knowledge of meaning and distribution of medicines can be converted into practical use. To our colleagues in medical anthropology we say that we should not hide in esoteric jargon or unintelligible 'thick description', but be prepared to write 'thinly' and transparently. The applicability of anthropological knowledge is a test for doing research.

• Let us, to conclude, give one example of how our understanding of the non-medical aspects of medicines may benefit our future health. We are referring to the arrival of antiviral medication in low-income countries.

## Antiretroviral medicines in low-income societies

The lowering of prices of antiretroviral therapy (ART) has led to a global commitment to scale up access to these drugs in resource poor settings, where until recently people living with HIV and AIDS (PLWA) did not have access to these life-prolonging drugs. Many different global actors have committed funds for rapid distribution of these drugs to people in need. The emphasis in the treatment programs is on the pharmacological efficacies of these drugs – they should be prescribed and used according to strict guidelines. Adherence has to be ensured, because non-adherence to ARV rapidly causes viruses to become resistant to the drugs, requiring the patient to switch to more expensive second-line therapies. The emergence of drug resistance is not only a problem to the individual, but also to society at large. It jeopardizes the treatment of all PLWA. There are many socio-cultural and psychological themes that require further studies, in addition to the biomedical studies done to optimise treatment regimes.

First, we need to understand how people understand the efficacy of ARVs. Preliminary fieldwork in resource poor settings suggests that users are initially very positive about the drugs, but are constrained by treatment related costs (including user-fees and transport costs). They tend to start taking the medicines when they are severely ill, and they experience rapid improvements in health [14-16]. However, many poor patients complain that the medicines cause hunger. Access to food becomes an issue that has not been sufficiently addressed in the treatment programs [14]. Also, some ARVs come with dietary restrictions (avoiding lipids, or taking ARVs at mealtime), which can be difficult for patients to comply with in family settings. The dietary restrictions can force them to disclose their HIV status – they need to explain why their food habits change [17]. Apart from the medical effects of the drugs, there is a need to understand such social (side) effects. There are not much data yet on long-term use of ARVs in resource poor settings [18,19]. ARV combinations can cause changes in fat-distribution in the body, gastro-intestinal disturbances and liver problems. These side-effects affect quality of life and can be stigmatising [20]. When after having taken ARVs for a few months, patients feel better, they may be less inclined to tolerate these (social) side-effects, resulting in treatment interruptions or treatment discontinuation.

Another issue for anthropological research is the emergence of many neo-traditional drugs for the prevention and treatment of AIDS. These drugs emerged when ARVs as life-prolonging treatment were not yet on accessible to poor users. They operated in the treatment vacuum. Now health workers advice patients not to use traditional medicines when taking ARVs, because of possible pharmacological interactions. Patients however consider these neo-traditional medicines as complementary or even alternative to modern pharmaceuticals. More details on actual use of remedies by PLWA is an important issue for further research, but sensitive because of the health workers rejection of these other treatments.

ARVs are drugs with a high monetary value. This makes their distribution and use an especially interesting theme for medical anthropologists. What happens with these valuable goods in the hands of people procuring, distributing, prescribing, dispensing and using them. Policy makers are aware of this high value of the drugs, and they are trying to control the distribution by means of complicated reporting systems, but to what extent will this disciplining attempt succeed? Paper is patient, and reports can be made about drugs supplies and prescriptions, when in reality different things are going on. Such leakage of essential medicines has been poorly studied in the past. Stock-outs are common with essential drugs, and they are likely to occur to with ARVs. The problem being that for ARVs the public health consequences are dramatic – it will contribute to rapid emergence of resistances. Anthropologists need to start studying the social lives of these valuable things.

## Competing interests

The author(s) declare that they have no competing interests.
